# Readiness of big health data analytics by technology-organization-environment (TOE) framework in Ethiopian health sectors

**DOI:** 10.1016/j.heliyon.2024.e38570

**Published:** 2024-09-27

**Authors:** Bayou Tilahun Assaye, Bekalu Endalew, Maru Meseret Tadele, Gizaw hailiye Teferie, Abraham Teym, Yidersal hune Melese, Andualem fentahun senishaw, Sisay Maru Wubante, Habtamu Setegn Ngusie, Aysheshim Belaineh Haimanot

**Affiliations:** a Department of Health Informatics, College of Medicine and Health Science, Debre Markos University, Debre Markos, Ethiopia; b Department of Public Health, College of Medicine and Health Science, Debre Markos University, Debre Markos, Ethiopia; c Department of Environmental Health, College of Medicine and Health Science, Debre Markos University, Debre Markos, Ethiopia; d Department of Human Nutrition, College of Medicine and Health Science, Debre Markos University, Debre Markos, Ethiopia; e Department of Health Informatics, College of Medicine and Health Science, University of Gondar, Gondar, Ethiopia; f Department of Health Informatics, College of Medicine and Health Science, Woldia University, Woldia, Ethiopia

**Keywords:** Big health data, Data analytics, Data management, Health information revolution, Health sectors, Readiness

## Abstract

**Background:**

Big health data is a large and complex dataset that the health sector has collected and stored continuously to generate healthcare evidence for intervening the future healthcare uncertainty. However, data use for decision-making practices has been significantly low in developing countries, especially in Ethiopia. Hence, it is critical to ascertain which elements influence the health sector's decision to adopt big health data analytics in health sectors. The aim of this study was to identify the level of readiness for big health data analytics and its associated factors in healthcare sectors.

**Methods:**

A cross-sectional study design was conducted among 845 target employees using the structural equation modeling approach by using technological, organizational, and environmental (TOE) frameworks. The target population of the study was health sector managers, directors, team leaders, healthcare planning officers, ICT/IT managers, and health professionals. For data analysis, exploratory factor analysis using SPSS 20.0 and structural equation modeling using AMOS software were used.

**Result:**

58.85 % of the study participants had big health data analytics readiness. Complexity (CX), Top management support (TMS), training (TR) and government law policies and legislation (GLAL) and government IT policies (GITP) had positive direct effect, compatibility (CT), and optimism (OP) had negative direct effect on BD readiness (BDR)

**Conclusion:**

The technological, organizational, and environmental factors significantly contributed to big health data readiness in the healthcare sector. The Complexity, compatibility, optimism, Top management support, training (TR) and government law and IT policies (GITP) had effect on big health data analytics readiness. Formulating efficient reform in healthcare sectors, especially for evidence-based decision-making and jointly working with stakeholders will be more relevant for effective implementation of big health data analytics in healthcare sectors.

## 1 Introduction

Big health data analytics is the process of extracting insights and generating knowledge from the vast amounts of data to improve patient outcomes, reduce costs, and enhance the overall quality of healthcare delivery [[1], [2], [3], [4]]. The healthcare industry has witnessed on an exponential growth in the volume, variety, velocity and quality of healthcare data [[5], [6], [7], [8]]. Even though, big health data analytics requires a high level of readiness, including the need for appropriate data infrastructure, skilled personnel and Different analytical tools [6,9].

The health information system is one of the main pillars of the Ethiopian health system's building blocks [10]. It is a thorough framework for organizing, processing, and evaluating health-related data in order to assist in the provision of healthcare services, public health surveillance health research and policy-making [11]. However, the health sector faces challenges in generating healthcare evidence and leveraging health data as a strategic asset to drive improvements in healthcare delivery and public health outcomes [12,13]. Researchers suggested that the health sector has paid more attention to the replacement of traditional approaches [[14], [15], [16]]. To handle the increasing amount of patient data, the healthcare sector should better prepare themselves to manage their huge data to derive important health trends, support timely preventive care, and facilitate the search for medical solutions [17,18]. A developing country like Ethiopia did not have clear information about its big health data analytics readiness for its implementation [19,20].

In the era of digitization, healthcare sectors are struggling to implement big data analytics for evidence-based planning in developing countries due to the growth of healthcare data [21,22]. However, those healthcare institutions are facing several challenges [23,24]. The first challenge is a lack of appropriate data infrastructure, including data storage, integration, security and fragmented data systems [6], which can be costly and time-consuming to implement [25]. Getting skilled personnel for big healthcare data analytics with a diverse set of skills, including data science, statistics, machine learning, and domain knowledge is other challenge [26], which makes it difficult for leveraging health data analytics to have a high impact on improving patient outcomes [27,28].

The scholar's findings show that organizational and cultural factors hinder the adoption and implementation of analytics initiatives, which makes it difficult to collaborate and share data across healthcare facilities [[29], [30], [31]]. The institution readiness level determines the success of big health data analytics initiatives that implement data-driven strategies to make informed healthcare decisions [32]. Identifying patterns and trends from the patient data helps to develop personalized treatment plans based on patient needs. Those who are at high risk of developing a particular condition also support healthcare providers in intervening early and preventing the onset of the disease through unnecessary tests and procedures [[33], [34], [35], [36], [37]].

Technology, environment, and organization are the major frameworks to assess the readiness of huge data analytics [38]that will enhance the healthcare sector's health information revolution for evidence-based decision-making [39]. Availability of appropriate technology, data infrastructure, analytical tools, and data governance policies that need to handle the volume, variety, and velocity of health data by ensuring the security, privacy, and ethical use of health data [40]. The environment framework creates an effect on the culture and structure of the healthcare sectors for data-driven decision-making and encourage collaboration across health facilities to adopt agile structure changes in the healthcare industry [41,42].

The organizational framework encompasses the availability of skilled personnel with expertise to invest in training and development health programs to build a team of diverse sets of skills, including data science, statistics, and machine learning professionals, to enhance their data management practices and improve healthcare efficiency [[43], [44], [45]]. Therefore, it is critical to investigate the current state of healthcare sector readiness for big health data analytics by technological, organizational, and environmental frameworks using a structural modeling approach. The following research questions were incorporated into this study.

• What are the technological factors influencing the readiness of big health data analytics in Ethiopian health sectors?
• How does the organizational structure affect the readiness of big health data analytics in the Ethiopian health sector?
• What are the environmental factors affecting the readiness of big health data analytics among study participants in Ethiopian health sectors?
• Are the study participants ready for big health data analytics?

## 2 Theoretical model

The TOE (technological, organizational, and environmental) framework enables us to understand how new technology adoption occurs [46]. Early technological adoption at the individual and organizational level helps to customize the system easily after assessing its compatibility and complexity [47]. Getting support for the adoption of new technology is critical for the integration of organizational services for better delivery and to take the risks associated with innovation adoption [[48], [49], [50], [51]].

Lastly, organizations may be encouraged or discouraged from implementing technology in an environmental setting by government laws, regulations, and IT policies [52,53].

### 2.1 TOE framework and hypothesis development

This research model indicated that there are relationships between technology, organization, and environmental conditions [54,55]. The compatibility, complexity, and optimism were hypothesized technological variables to have major effects on the big health data analytics readiness. Upper management support, financial support and training [56] are thought to have an effect on the preparedness of health sectors towards big health data analytics [57]. The government's IT policies, regulations, and legislation were hypothesized to have a significant impact on big health data analytics readiness [58]. The following general hypothesis was suggested based on the general research question of the study to test the readiness of big health data analytics in the health sector.

1. There is readiness difference to analysis big health data among healthcare sector in the study area.
2. The readiness to analysis big health data in the healthcare sectors of the study area is affected by a certain predictors.

### 2.2 Technology context

The complexity, compatibility, and optimization features influences on the readiness of big health data analytics and on its implementations [[60], [61], [62]]. Recent research on the function of complexity has discovered a detrimental effect on the implementation of big health data analytics [56,63]. Having a favorable attitude towards a new technology means having a solid view of how to improve employees' daily lives in terms of managing their work efficiently [[64], [65], [66]]. Compatibility assesses the degree to which a new system is compatible with the organization's culture and business processes [59,67], which has been a crucial factor in driving technology adoption [67]. The following hypothesis was suggested to test the readiness of big health data analytics in the health sectors.

Hypothesis 1 (H1) Complexity has an effect on big health data analytics readiness in the healthcare sector.

Hypothesis 2 (H2) Compatibility has an effect on big health data analytics readiness in the healthcare sector.

Hypothesis 3 (H3) Optimism has an effect on big health data analytics readiness in the healthcare sector.

### 2.3 Organizational context

Organizational preparedness and management support were factors affecting the healthcare sector's readiness for big health data analytics. It has been the extent to which managers grasp and embrace new technology [68,69]. To capitalize on big health data analytics, businesses must execute a set of procedures that require funding to supply the necessary human and material resources.

Financial support is other organizational aspect that is critical to the acquisition of a new system, as are payment incentives and infrastructure security [70]. Continuous learning enables individuals to share information with others and healthcare sector employees require formal training for all staff in order to acquire new skills necessary to perform their jobs [71]. The following hypothesis was suggested to test the readiness of big health data analytics in the health sectors.

Hypothesis 4 (H4) Top management support has an effect on big health data analytics readiness in the healthcare sector.

Hypothesis 5 (H5) Financial support has an effect on big health data analytics in the healthcare sector.

Hypothesis 6 (H6) Training has an effect on big health data analytics readiness in the healthcare sector.

#### 2.3.1 Environmental context

The TOE model helps identify the effects of government laws and policies as external factors that influence the adoption of big health data analytics. The government's IT policy and government laws and legislation are critical for innovation uptake [72], and government rules have also been identified as significant drivers affecting the adoption of novel technologies, particularly in developing nations [73]. The following hypothesis was suggested to test the readiness of big health data analytics in the health sectors.

Hypothesis 7 (H7) Government IT policies have an effect on big health data analytics readiness in the healthcare sector.

Hypothesis 8 (H8) Government lows and legislations have an effect on big health data analytics readiness in the healthcare sector.

## 3 Methods and materials

### 3.1 Study design

An institution-based cross-sectional study design method was conducted.

### 3.2 Study area and Period

This study was conducted among employees working public health sectors in Ethiopia from 2022 August to June 2023.

### 3.3 Source and study populations

#### 3.3.1 Source population

All employees working at public health sectors in Ethiopia were the source population.

#### 3.3.2 Study population

The study populations of this study were health sector managers, directors, team leaders/coordinators, healthcare M&E/planning officers, ICT/IT managers, and health professionals who were working in public health sectors, which were found in Ethiopia.

### 3.4 Inclusion and exclusion criteria

All employees who had been working for at least six months in the public health sector were included in the study. However, employees who were working for less than six months, seriously ill, and unable to respond were excluded from the study.

### 3.5 Sample size and sampling procedures

#### 3.5.1 Sample size determination

The sample size was determined by using a single population proportion formula of 50 % employees' proportions because we couldn't find any study conducted to determine the readiness of big health data analytics, and then multistage sampling techniques were employed by considering a 10 % non-response rate and with the design effect 2. Were n1 = is calculated sample size, Z = confidence interval [95 %], P = proportion of readiness among employees = 50 %, 1-p = proportion of not ready among employees, d = marginal error [5 %], none response rate of 10 %, design effect = 2.

**Figure:**
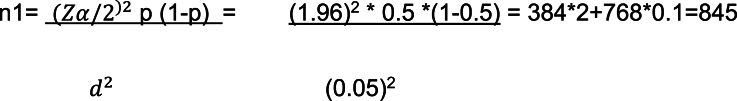

### 3.6 Operational definitions

**Big health data:** The amount of data that can be measured in its volume, variety, velocity, veracity (quantity of data, different forms or types of data, measure of how fast data is flowing, inconsistencies and uncertainty of health data) respectively [[74], [75], [76], [77]].

### 3.7 Big health data analytics

Health sectors, starting from data collection to data synthesizing evidence generation, such as data comparison, estimation, prediction of future trends, testing the quality of data, and identifying healthcare gaps in the health system by using different application software [[78], [79], [80], [81]].

### 3.8 Data collection procedures and data quality assurance

#### 3.8.1 Data collection procedures

The goal of this study was to construct a model that will help health sectors in Ethiopia, a developing country, understand the linkages between technology, organization, and environmental (TOE) settings and BD readiness in healthcare sectors to use BD in healthcare.

The questionnaire was adapted and measured on a five-point Likert scale ranging from “1” to “5”. i.e., ‘1’ for very disagree, 2′ for disagree,3′ for neutral, ‘4’ for agree, and ‘5’ for very agree, were used. One can score a minimum of ‘45’ and a maximum of ‘225’ related to measuring readiness study participants towards big data health analytics.

#### 3.8.2 Data quality assurance

Before the actual data collection, a pre-test was done on 5 % of the study participants, and modifications were made based on the pre-test. The data collectors and the supervisor were selected and given training before participating in the data collection process. Creating awareness to the respondents about the purpose of the study, their rights, and sufficient time was given to respondents for reading and filling out materials carefully. There was continuous supervision up to the end of data collection. After the collection of data, the supervisor and the investigator assured the quality of the data by removing noise and inconsistencies, filtering out unwanted outliers and missing values, and checked out its consistency and completeness to make sure there were more accurate and reliable results.

#### 3.8.3 Data processing and analysis

**Fig. 1:**
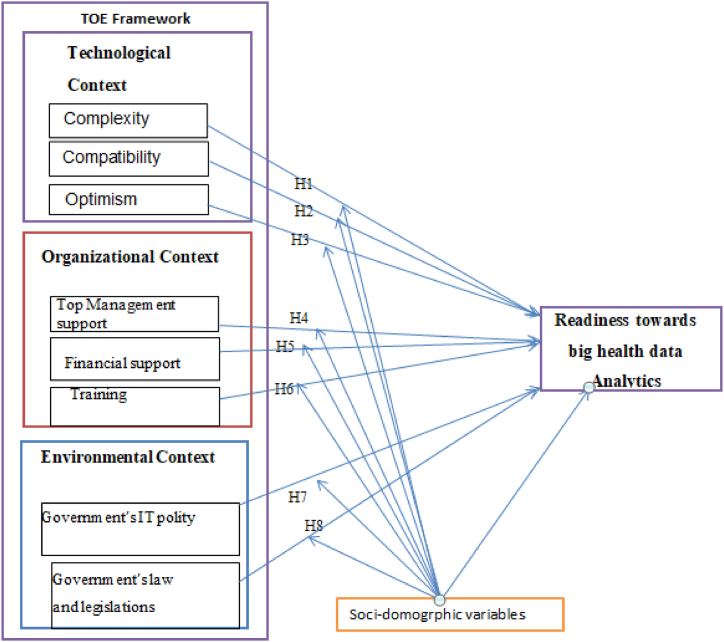
TOE conceptual framework for the readiness of big health data analytics in Health Sectors, Ethiopia, 2023 [58,59].

All data were analyzed using AMOS and SPSS. Descriptive statistics such as mean with standard deviation and median with inter-quartile range (IQR) were used for numerical variables. In addition, to make the constructed mean score comparable and standardized, we first transformed the observed mean score into a range (0–100). Frequencies and percentages were employed for categorical variables. Following descriptive data exploration, confirmatory factor analysis (CFA) was performed to determine whether or not the factor loading on the individual items was considerable. Finally, SEM was used to analyze the direct and indirect effects of variables on the preparation of employees for big data analytics (see Fig. 1).

**Fig. 2:**
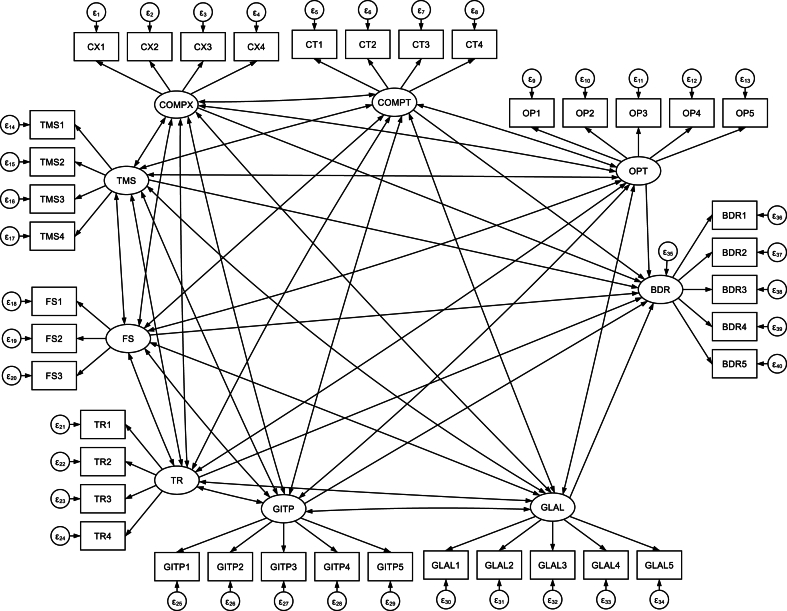
Theoretical model of big health data analytics readiness among healthcare employees in Ethiopia, 2023.

The SEM has two components: the measurement model and the structural model. The structural components indicate the relationship between the latent variables; the common bias was assessed through variance inflation factors (VIF) values of the inner model. In this study all the VIF values are lower than 3.3, the model of study was considered free from of common method bias, whereas the measurement components evaluate the relationship between a latent variable and its items. The analysis began with the theoretical model (Fig. 2) and iterative modifications were made by adding paths if theoretically supported, and comparing by considering different measure of the model fitness indexes such as Root Mean Squared Error Approximation (RMSA), Goodness of Fit Index (GFI), Normed Fit Index (NFI), Tucker Lewis Index (TLI), and Comparative Fit Index (CFI) and measure of model parsimony such as information criteria (AIC and BIC).

**Fig. 3:**
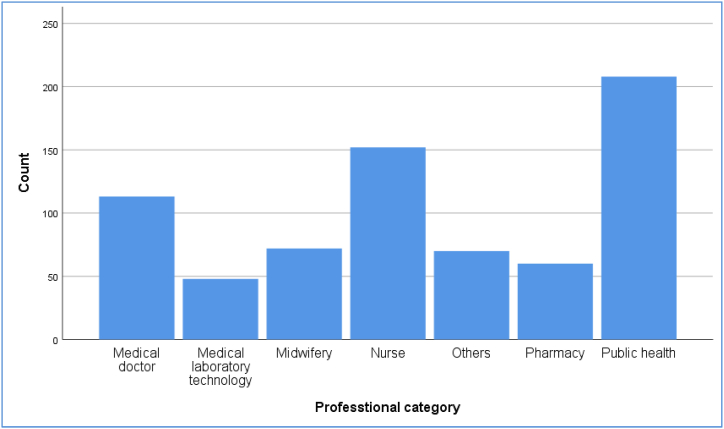
Employee's professions category in health sectors, Ethiopia, 2023.

Finally, an over-identified model with an RMSEA close to 0.05 and the least information criterion was retained. The A single-headed arrows and their respective path coefficients diagrammatically depicted the influence of each exogenous or mediating variable on the respective dependent variable, whereas double-headed arrows and their respective path coefficients showed the correlation among error terms as well as residuals of constructs. A 95 % confidence interval and a p-value less than 0.05 were used to evaluate statistical significance.

## 4 Result

Structural equation modeling was used in this study to analyze the factors that affect big health data analytics readiness to improve healthcare service delivery in the Ethiopian healthcare sector. The data results show that the healthcare sector's readiness for big health data analytics had a significant impact on the healthcare sector in Ethiopia.

### 4.1 Socio demographic variables

Before evaluating the developed model, descriptive statistics about the study participants' backgrounds were analyzed. Two-thirds (479) of the 723 participants' (66.25 %) were men. Their average age was 33 years, with a 7-year standard deviation (SD). Their median work experience was nine years, with an IQR of 8 (13-5) years. Most of the research participants, 414 (57.26 %), had a bachelor's degree. Almost one-quarter of all participants, 183 (25.31 %), had taken basic computer system training. About half, 367 (50.76 %) of them had no computer access, whereas more than three-fourths 628 (86.86 %) of them had internet access. In terms of internet usage, one-seventh of 107 (14.80 %) participants never utilized the internet. The Analysis results are given in Table 1.

**Table 1:** Background characteristics of participants’ in health sectors, Ethiopia, 2023.

Variable	Category	Number	Percentage
Sex of the respondent	Male	479	66.25
	Female	244	33.75
Age	Mean ± SD	33.03 ± 7.02	
Employee's Profession	Medical doctor	113	15.63
	Public health	208	28.77
	Pharmacy	60	8.30
	Midwifery	72	9.96
	Nurse	152	21.02
	Medical laboratory	48	6.64
	Others 1	70	9.68
Educational status	Diploma	90	12.45
	Bachelor's degree	414	57.26
	Master's degree	100	13.83
	Medical doctor	115	15.91
	Others 2	4	0.55
Work experience	Median ± IQR	9 ± 8 (13-5)	
Computer system training	Never attended basic computer training	183	25.31
	Just have introductory level	250	34.58
	Have a diploma	100	13.83
	BSC in the ICT areas	60	8.30
	Certified in the ICT	124	17.15
	Others 3	6	0.83
Take training	Yes	419	57.95
	No	304	42.05
Computer access	Yes	356	49.24
	No	367	50.76
Internet access	Yes	628	86.86
	No	95	13.14
Internet utilization	Never	107	14.80
	Rarely	46	6.36
	Sometimes	304	42.05
	Often	64	8.85
	Always	202	27.94

Others1, ICT/IT, HI, HIT and Environmental health; Others2, Diploma, PhD; Others3, Masters.

Regarding the employees profession type, the participated in this study are public health 208 (28.77 %), medical doctor 113 (15.63 %), nurse 152 (21.02 %), midwifery 72 (9.96 %), pharmacy 60 (8.30 %) and medical laboratory 48 (6.64 %) (Fig. 3).

### 4.2 Magnitude of constructs in the model

Among the constructs, respondents scored highest and lowest mean score in optimism (75.61 with 16.78 SD) and BDR (58.85 with 18.89 SD), respectively (Table 2).

**Table 2:** Magnitude of BDR among Employees in health sectors, Ethiopia, 2023.

Variable	N	Mean	SD	SE	95%CI	Minimum	Maximum
Complexity	723	63.52	18.30	0.68	(62.18, 64.86)	12.5	100
Compatibility	723	62.50	18.12	0.67	(61.18, 63.82)	25	100
Optimism	723	75.61	16.78	0.62	(74.38, 76.83)	15	100
TMS	723	63.96	19.41	0.72	(62.54, 65.38)	18.75	100
FS	723	69.25	19.08	0.71	(67.86, 70.64)	25	100
Training	723	61.12	19.76	0.73	(59.67, 62.56)	18.75	100
GITP	723	60.70	18.24	0.68	(59.37, 62.03)	20	100
GLAL	723	59.88	18.79	0.70	(58.50, 61.25)	15	100
BDR	723	58.85	18.89	0.70	(57.47, 60.23)	10	100

### 4.3 Internal consistency and correlations between the latent variables

Cronbach's alpha was determined for each latent variable in the instrument to ensure internal consistency. Cronbach's alpha values for all latent variables were high (>0.7). Furthermore, the tool's overall internal consistency was calculated (α = 0.932). Inter-latent correlation revealed a statistically significant relationship between constructs; there is a moderate positive correlation between GLAL and GITP (r = 0.623, p 0.001) and between GLAL and BDR (r = 0.650, p 0.001), as opposed to complexity and compatibility, which had a relatively weak positive correlation (r = 0.052, p 0.001) (Table 3).

**Table 3:** Internal consistency and correlations between the latent variables.

Constructs	Cronbach's alpha	Complexity	Compatibility	Optimism	TMS	FS	TR	GITP	GLAL	BDR
Complexity	0.702	1								
Compatibility	0.722	0.052	1							
Optimism	0.838	0.454	0.450	1						
TMS	0.758	0.429	0.406	0.366	1					
FS	0.715	0.329	0.360	0.466	0.611	1				
TR	0.740	0.272	0.253	0.329	0.343	0.359	1			
GITP	0.832	0.333	0.401	0.452	0.401	0.374	0.398	1		
GLAL	0.834	0.405	0.390	0.413	0.465	0.430	0.384	0.623	1	
BDR	0.788	0.403	0.332	0.255	0.443	0.328	0.429	0.458	0.650	1

All results are Pearson's correlation matrices significant at 5 % level of significance.

### 4.4 Sample size adequacy and sphericity

We used the Kaiser-Meyer-Olkin (KMO) measure of sampling adequacy to examine the sample size adequacy for each construct individually as well as collectively (KMO>0.7, p-value<0.001). Table 4 displays the results of the KMO and Bartlett's test.

**Table 4:** The KMO test of the nine constructs individually and collectively (all 9 constructs together).

Construct	COMPX	COMPT	OPT	TMS	FS	TR	GITP	GLAL	BDR	Overall
KMO	0.707	0.738	0.838	0.755	0.721	0.734	0.803	0.812	0.793	0.898
P-value	<0.001	<0.001	<0.001	<0.001	<0.001	<0.001	<0.001	<0.001	<0.001	<0.001

### 4.5 Confirmatory factor analysis

**Fig. 4:**
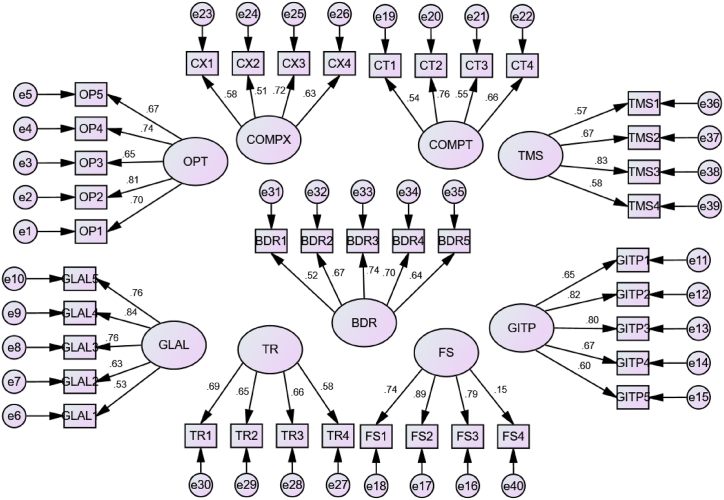
Measurement model for each constructs with their standardized estimates displayed.

In this study, we performed CFA for all constructs and then tested the key assumption, which states that the factor loading for each construct on the respective items should be more than 0.5. Except for FS on item 4 (loading = 0.15), all other factor loadings were more than 0.5. As a result, we eliminated the fourth item from the FS construct from the final model and rechecked the CFA for FS with the remaining three items, which passed the assumption (all loadings >0.5). The CFA results for all constructs, together with their factor loadings, are shown in (Fig. 4) below.

### 4.6 Factors associated with BDR

**Fig. 5:**
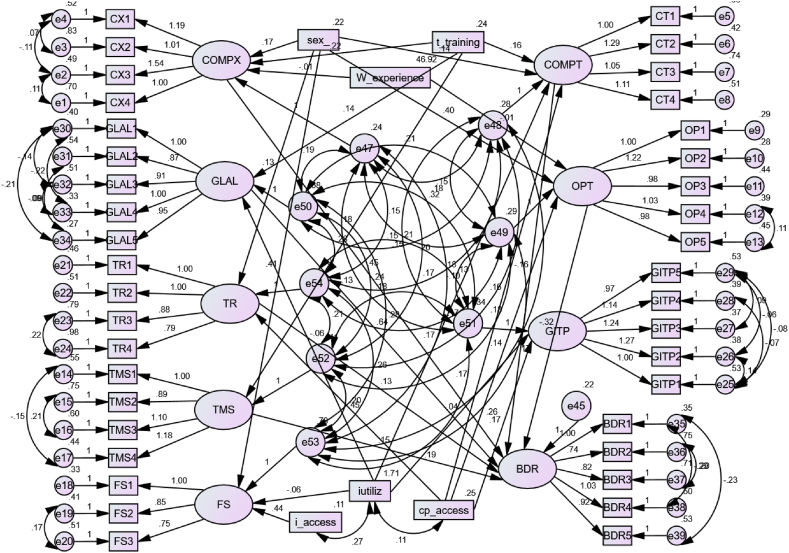
SEM for factors associated with BDR among healthcare employees in, Ethiopia healthcare sectors, 2023.

Fig. 5 depicts the final fitted model, which includes both the structural and measurement components of structural equation modeling. This model had better model fitness indices (CMIN/DF = 3.778, RMR = 0.092, GFI = 0.833, TLI = 0.804, CFI = 0.827, and RMSEA = 0.062) and lower Akaike information criteria and Bayesian information criterion values when compared to other fitted models; hence, it was chosen as the relatively appropriate model. Some factors, such as profession and educational status, were left out of the final model because their estimated contributions were not statistically significant at a 0.05 alpha level. Furthermore, due to a multicollinearity issue with job experience, the variable age is also omitted from the final model.

This model included six exogenous observed variables (sex, work experience, computer access, internet access, internet utilization, and whether or not they took training), nine endogenous unobserved variables (complexity (CX), compatibility (CT), optimism (OP), top management support (TMS), financial support (FS), training (TR), government IT policies (GITP), government policies and legislation (GLAL), and BD readiness (BDR) in healthcare). At an alpha level of 0.05, all of the path coefficients in the figure were statistically significant in the final model. Due to a relatively low loading contribution on the latent variable, we removed one of the financial support items at CFA. The exogenous observed variables, namely work experience, sex, take training, computer access, and internet utilization, were related to BDR indirectly but not directly via the mediator variables (COMPX and OPT); (OPT, TR, and COMPX); (TMS, GLAL, COMPX, and OPT); and (GLAL and COMPT), respectively.

Specifically, complexity (adjusted β = 0.297, 95%CI; 0.172, 0.494), TMS (adjusted β = 0.123, 95%CI; 0.022, 0.224), TR (adjusted β = 0.123, 95%CI; 0.027, 0.226), and GLAL (adjusted β = 0.635, 95%CI; 0.551, 0.721) had a positive direct effect on BDR. Moreover, training (adjusted β = 0.121, 95%CI; 0.073, 0.176) had an indirect positive effect on BDR. However, COMPT (adjusted β = −0.116, 95%CI; −0.269, −0.001), OPT (adjusted β = −0.116, 95%CI; −0.269, −0.001) had a direct negative effect on BDR. Moreover, sex (adjusted β = −0.036, 95%CI; −0.075, −0.003), work experience (adjusted β = −0.006, 95%CI; −0.048, 0.021), computer access (adjusted β = −0.029, 95%CI; −0.061, 0.002), and internet utilization (adjusted β = −0.038, 95%CI; −0.089, 0.012) had an indirect negative effect on the BDR via the mediator variable complexity, (complexity and optimism), TR, and (GLAL and COMPT), respectively. In addition, sex (adjusted β = 0.156, 95%CI; 0.081, 0.227) and training (adjusted β = 0.156, 95%CI; 0.081, 0.227) on complexity; sex (adjusted β = 0.116, 95%CI; 0.047, 0.187), training (adjusted β = 0.146, 95%CI; 0.070, 0.224), and computer access (adjusted β = 0.144, 95%CI; 0.071, 0.216) on COMPT; sex (adjusted β = 0.328, 95%CI; 0.263, 0.386) and computer access (adjusted β = 0.121, 95%CI; 0.057, 0.181) on OPT; training (adjusted β = 0.136, 95%CI; 0.072, 0.200) on TMS; sex (adjusted β = 0.215, 95%CI; (0.152, 0.276) and internet access (adjusted β = 0.165, 95%CI; 0.091) on FS; sex (adjusted β = 0.086, 95%CI; 0.010, 0.276) and computer access (adjusted β = 0.138, 95%CI; 0.065, 0.208) on TR; computer access (adjusted β = 0.145, 95%CI; 0.078, 0.211) on GITP, and take training (adjusted β = 0.092, 95%CI; 0.035, 0.147) on GLAL had a direct positive effect whereas work experience (adjusted β = −0.112, 95%CI; −0.197, −0.031) on COMPX; internet utilization (adjusted β = −0.231, 95%CI; −0.294, −0.163) on COMPT; work experience (adjusted β = −0.114, 95%CI; −0.179, −0.056) on OPT; internet utilization (adjusted β = −0.080, 95%CI; −0.154) on FS; internet utilization (adjusted β = −0.085, 95%CI; −0.163, −0.010) on GITP, and internet utilization (adjusted β = −0.103, 95%CI; −0.166, −0.037) on GLAL, had a direct negative effect (Table 5 and Fig. 5).

**Table 5:** The direct, indirect, and total effects of socio-demographic factors and technology, organization and environment context on big data readiness among healthcare employees in Ethiopia, 2023.

	Variables	Category	Direct effect(95%CI)	Indirect effect(95%CI)	Total effect(95%CI)
**DVs**	**IVs**				
BDR	Complexity		0.297(0.172, 0.494)	–	–
	Compatibility		−0.116(-0.269, −0.001)	–	–
	Optimism		−0.242(-0.334, −0.166)	–	–
	TMS		0.123(0.022, 0.224)	–	–
	TR		0.123(0.027, 0.226)	–	–
	GITP		0.623(0.542–0.680)	–	–
	GLAL		0.635(0.551, 0.721)	–	–
	Sex	Male	–	−0.036(-0.075, −0.003)	−0.036(-0.075, −0.003)
		Female	0	0	0
	Work experience		–	−0.006(-0.048, 0.021)	−0.006(-0.048, 0.021)
	Take training	Yes	–	0.121(0.073, 0.176)	0.121(0.073, 0.176)
		No	0	0	0
	Computer access	Yes	–	−0.029(-0.061, 0.002)	−0.029(-0.061, 0.002)
		No	0	0	0
	Internet Utilization		–	−0.038(-0.089, 0.012)	−0.038(-0.089, 0.012)
Complexity	Sex	Male	0.156(0.081, 0.227)	–	–
		Female	0	0	0
	Work experience		−0.112(-0.197, −0.031)		–
	Take training	Yes	0.211(0.137, 0.294)	–	–
		No	0	0	0
Compatibility	Sex	Male	0.116(0.047, 0.187)	–	–
		Female	0	0	0
	Take training	Yes	0.146(0.070, 0.224)	–	–
		No	0	0	0
	Internet Utilization		−0.231(-0.294, −0.163)	–	–
	Computer access	Yes	0.144(0.071, 0.216)	–	–
		No	0	0	0
Optimism	Sex	Male	0.328(0.263, 0.386)	–	–
		Female	0	0	0
	Work experience		−0.114(-0.179, −0.056)	–	–
	Computer access	Yes	0.121(0.057, 0.181)	–	–
		No	0	0	0
TMS	Take training	Yes	0.136(0.072, 0.200)	–	–
		No	0	0	0
FS	Sex	Male	0.215(0.152)	–	–
		Female	0	0	0
	Internet access	Yes	0.165(0.091)	–	–
		No	0	0	0
	Internet Utilization		−0.080(-0.154)	–	–
TR	Sex	Male	0.086(0.010, 0.276)	–	–
		Female	0	0	0
	Computer access	Yes	0.138(0.065, 0.208)	–	–
		No	0	0	0
GITP	Computer access	Yes	0.145(0.078, 0.211)	–	–
		No	0	0	0
	Internet Utilization		−0.085(-0.163, −0.010)	–	–
GLAL	Take training	Yes	0.092(0.035, 0.147)	–	–
		No	0	0	0
	Internet utilization		−0.103(-0.166, −0.037)	–	–

Key: DV = Dependent Variable.

## 5 Discussion

In the era of digitalization, health sectors are focusing and contextualizing their plans for healthcare service delivery based on big data for future healthcare interventions. However, implementing big data analytics needs intensive resources to examine the influence of technology, organization, and environmental factors to generate healthcare evidence [1,14,23]. The increasing volume, velocity, and variety of data generated in the current health system can make it difficult for organizations to effectively collect, store, process, and analyze the data [14,15,82].

According to the findings, complexity issues were found to be critical issues for the acceptance of big data analytics, which revealed that there was a positive direct influence on adopting big data analytics. Most prior scholars demonstrated that complexity is a common challenge for developing countries to accept new technology and innovation. However, it boosts organizational performance across the healthcare sector [15,16,82].

This study also indicated that optimism had a direct negative effect on BDR. As the scholar suggested, the optimism of the users is the inducer of technical facilities that encourage accepting new technology. However, insecurity and discomfort serve as barriers that discourage the adoption of new technologies [83,84]. Compatibility also had a direct negative effect on the readiness of big health data technology. The result of this study is consistent with a study conducted on technology adoption [85,86], which contends that complexity and optimistic variables The inhibiting aspects of technological facilities shall operate separately [1,87].

This study revealed that environmental context factors significantly influence the adoption of big data analytics in the healthcare sectors [88] and the study will form the basis for examining how to generate healthcare evidence within the sphere of healthcare facilities [89,90].

Top management support has a significant effect on the adoption of big health data analytics [91]. However, some difficulties negate the applicability of big data in the health system. Financial support is key and essential for data collection, storage, transmission, analysis, and adoption [85,87,92]. To provide the necessary skilled data analysts and material resources, as well as to manage potential dangers, each process needs funds [86].

This research finding indicated that the government's IT policy and legalization have a significant influence on healthcare data analytics, which are provided by the government authority to encourage the assimilation of IT innovation [[93], [94], [95]]. Adoption of new technology may be significantly impacted by a country's laws and regulations. Government rules can either encourage or deter enterprises from implementing BD [29,96].

Most scholars suggested that understanding the value of data gained via analysis is mandatory rather than the accumulation of data [97]. Therefore, healthcare sectors need to increase employee training and recruit data talents to upgrade their sophisticated technology in real-time in order to implement the necessary BD adoption measures [98,99]. Investigating and putting into practice the aforementioned steps can improve technical capability, generate knowledge, increase healthcare productivity and profitability [100] and help future healthcare preparedness [101].

## 6 Conclusion

The technological, organizational, and environmental factors were significantly contributed towards big health data readiness in the healthcare sectors. The TOE framework components had a direct effect on the readiness of big health data analytics, such as complexity, compatibility, optimism, Top management support, training (TR) and government law and IT policies (GITP) had effect on big health data analytics readiness. Formulating efficient reform on healthcare sectors, especially for evidence-based decision-making and jointly working with stakeholders will more relevant for effective implementation of big health data analytics in healthcare sectors.

Therefore, the government should take the initiative to strengthen opportunities for health sectors and employees to learn and apply data analytics in their healthcare practice by providing ICT training, working with collaborators, and motivating the staff for healthcare management, especially for health information use in their health sectors.

## 7 Strengths and limitations of the study

The sample size was large which help to generalize the result. The result of this study used as a baseline for other researchers and helps the scalability of health sectors to data use and information sharing cultures. However, the study was not supported by a qualitative study, as we employed a simple random sampling method that may be affected by the sample frame and its lower precision. The study was conducted only at public health sectors that might affect the result.

## Ethical approval and consent to participate

Ethical clearance was obtained from Debre Markos University, College of medicine and health science with approval number ”**HSC/R/C/ser/PG/CO/420/12/15”**, and support letters from health bureaus. Written informed consent was obtained from each study participant after telling the objective of the study. All methods were performed in accordance with the relevant guidelines and regulations. The data collection procedure was anonymous.

## Consent for publication

Not applicable.

## Funding

The funding body has no role in the design of the study and collection, analysis, interpretation of the data, writing the manuscript, and publication as well.

## Guarantor declaration statement

Not Applicable.

## Data availability statement

The data associated with this study will be made available upon reasonable request**.**

## CRediT authorship contribution statement

**Bayou Tilahun Assaye:** Writing – review & editing, Writing – original draft, Visualization, Validation, Supervision, Software, Resources, Project administration, Methodology, Investigation, Funding acquisition, Formal analysis, Data curation. **Bekalu Endalew:** Formal analysis, Data curation. **Maru Meseret Tadele:** Investigation, Conceptualization. **Gizaw hailiye Teferie:** Supervision, Formal analysis. **Abraham Teym:** Investigation, Formal analysis, Data curation. **Yidersal hune Melese:** Formal analysis, Conceptualization. **Andualem fentahun senishaw:** Formal analysis, Data curation. **Sisay Maru Wubante:** Formal analysis, Data curation. **Habtamu Setegn Ngusie:** Writing – original draft, Software, Resources, Formal analysis. **Aysheshim Belaineh Haimanot:** Resources, Methodology, Formal analysis.

## Declaration of competing interest

The authors declare that they have no known competing financial interests or personal relationships that could have appeared to influence the work reported in this paper.

## Abbreviations

complexity: CX
compatibility: CT
optimism: OP
top management support: TMS
financial support: FS
training: TR
government IT policies: GITP
government policies and legislation: GLAL
and BD readiness: BDR
BSC: Bachelor of Science
E-Health: Electronic Health
GP: General Practitioners
HI: Health Informatics
HIT: Health Information Technician
HP: Health professionals
MPH: Master of Public Health
ICT: Information communication technology
IT: Information technology
SPSS: Statistical Package for Social Science
WHO: World Health Organization

## References

[1] Yves, H. and I. Moseti-Morara, A Technology, Organisation, Environment (TOE) Based Framework for Big Data Analytics (BDA) Adoption in Healthcare in African Countries*.* p. 133.

[2] Goswami C. (2024). Securing healthcare big data in industry 4.0: cryptography encryption with hybrid optimization algorithm for IoT applications. Opt. Quant. Electron..

[3] Al Zaabi M., Alhashmi S.M. (2024). Big data security and privacy in healthcare: a systematic review and future research directions. Inf. Dev..

[4] Sivarajah U. (2024). A study on big data analytics and innovation: from technological and business cycle perspectives. Technol. Forecast. Soc. Change.

[5] Dash S. (2019). Big data in healthcare: management, analysis and future prospects. Journal of big data.

[6] Rehman A., Naz S., Razzak I. (2022). Leveraging big data analytics in healthcare enhancement: trends, challenges and opportunities. Multimed. Syst..

[7] Gupta B.B. (2024).

[8] Adekugbe A.P., Ibeh C.V. (2024). Advancing healthcare data solutions: comparative analysis of business and research models in the US. International Medical Science Research Journal.

[9] Majdzadeh R. (2024). Big data revolution: transforming business landscapes through data-driven decision making. Social Sciences Spectrum.

[10] Worku K. (2023).

[11] Manyazewal T. (2017). Using the World Health Organization health system building blocks through survey of healthcare professionals to determine the performance of public healthcare facilities. Arch. Publ. Health.

[12] Taye G. (2021). The Ethiopian Health Information System: where are we? And where are we going?. Ethiop. J. Health Dev..

[13] Tilahun B. (2021). Strengthening the national health information system through a capacity-building and mentorship partnership (CBMP) programme: a health system and university partnership initiative in Ethiopia. Health Res. Pol. Syst..

[14] Austin C.C. (2018). 2018 IEEE International Conference on Big Data (Big Data).

[15] Vasilyeva O., Richardson A. (2022).

[16] Venkatraman S., Sundarraj R. (2023). Assessing organizational health-analytics readiness: artifacts based on elaborated action design method.

[17] Kalaiselvi V., Tripathy J.P. (2024). Principles and Application of Evidence-Based Public Health Practice.

[18] Rashid A. (2024). Big data analytics-artificial intelligence and sustainable performance through green supply chain practices in manufacturing firms of a developing country. Journal of Science and Technology Policy Management.

[19] Benda N. (2024). Designing electronic data capture systems for sustainability in low-resource settings: viewpoint with lessons learned from Ethiopia and Myanmar. JMIR Public Health and Surveillance.

[20] Regane, B., Job Market for Data Science and Big Data in East Africa.

[21] Khan R., Usman M., Moinuddin M. (2024). From raw data to actionable insights: navigating the world of data analytics. International Journal of Advanced Engineering Technologies and Innovations.

[22] Faridoon L., Liu W., Spence C. (2024). The impact of big data analytics on decision-making within the government sector. Big Data.

[23] Joubert A., Murawski M., Bick M.J.I.S.F. (2023). Measuring the big data readiness of developing countries–index development and its application to Africa.

[24] Asri H. (2015). 2015 International Conference on Cloud Technologies and Applications (CloudTech).

[25] Mneney J., Van Belle J.-P. (2016). 2016 6th International Conference-Cloud System and Big Data Engineering (Confluence).

[26] Abedjan Z. (2019).

[27] Yuen H.-W., Balakrishnan A. (2020). Big data in medical education–Are we ready?.

[28] Farouk F.M., Siew E.-G., Yusof S.H. (2024). Overcoming resistance to change in a big data analytics implementation case study. J. Inf. Technol. Teach. Cases.

[29] Yusif S., Hafeez-Baig A., Soar J. (2020).

[30] Barham H., Daim T.J.S.C. (2020).

[31] Raghupathi W., Raghupathi V.J.H.i.s. (2014).

[32] Kalema B.M., Busobozi V.V., Engineering i.A.i.S. (2020).

[33] Pastorino R. (2019). Benefits and challenges of Big Data in healthcare: an overview of the European initiatives.

[34] Batko K., Ślęzak A. (2022). The use of Big Data Analytics in healthcare.

[35] Krishnan S.M. (2016). 2016 32nd Southern Biomedical Engineering Conference (SBEC).

[36] Shahbaz M. (2019). Investigating the adoption of big data analytics in healthcare: the moderating role of resistance to change.

[37] Kankanhalli A. (2016).

[38] Badghish S., Soomro Y.A. (2024). Artificial intelligence adoption by SMEs to achieve sustainable business performance: application of technology–organization–environment framework. Sustainability.

[39] Gomes M.A.S. (2023). Transforming healthcare with big data analytics: technologies, techniques and prospects.

[40] Raj P. (2015).

[41] Galetsi P., Katsaliaki K., Kumar S. (2020). Big data analytics in health sector: theoretical framework, techniques and prospects.

[42] Youssef A.E. (2014). A framework for secure healthcare systems based on big data analytics in mobile cloud computing environments.

[43] Palanisamy V., Thirunavukarasu R., Sciences I. (2019). Implications of big data analytics in developing healthcare frameworks–. A review.

[44] Mohamed A. (2020). The state of the art and taxonomy of big data analytics: view from new big data framework.

[45] Kruse C.S. (2016). Challenges and opportunities of big data in health care: a systematic review.

[46] Baker J. (2012). The technology–organization–environment framework. Information Systems Theory: Explaining and Predicting Our Digital Society.

[47] Awa H.O., Ukoha O., Emecheta B.C. (2016). Using TOE theoretical framework to study the adoption of ERP solution. Cogent Business & Management.

[48] Nkhoma M.Z., Dang D., De Souza-Daw A. (2013). Proceedings of the European Conference on Information Management & Evaluation.

[49] England I., Stewart D. (2007). Executive management and IT innovation in health: identifying the barriers to adoption. Health Inf. J..

[50] Gopalakrishna-Remani V., Jones R.P., Camp K.M. (2019). Levels of EMR adoption in US hospitals: an empirical examination of absorptive capacity, institutional pressures, top management beliefs, and participation. Inf. Syst. Front.

[51] Amini M., Jahanbakhsh Javid N. (2023). *A multi-perspective framework established on diffusion of innovation (DOI) theory and technology, organization and environment (TOE) framework toward supply chain management system based on cloud computing technology for small and medium enterprises.* Organization and Environment (TOE) Framework toward Supply Chain Management System Based on Cloud Computing Technology for Small and Medium Enterprises (January 2023). International Journal of Information Technology and Innovation Adoption.

[52] Zhu K., Kraemer K.L., Dedrick J. (2004). Information technology payoff in e-business environments: an international perspective on value creation of e-business in the financial services industry. J. Manag. Inf. Syst..

[53] Blind K. (2016). Handbook of Innovation Policy Impact.

[54] Yeh C.-H., Lee G.-G., Pai J.-C. (2015). Using a technology-organization-environment framework to investigate the factors influencing e-business information technology capabilities. Inf. Dev..

[55] Lippert S.K., Govindarajulu C. (2006). Technological, organizational, and environmental antecedents to web services adoption. Communications of the IIMA.

[56] Lai Y., Sun H., Ren J. (2018). Understanding the determinants of big data analytics (BDA) adoption in logistics and supply chain management: an empirical investigation. Int. J. Logist. Manag..

[57] Lutfi A. (2022). Factors influencing the adoption of big data analytics in the digital transformation era: case study of Jordanian SMEs. Sustainability.

[58] Ghaleb E.A. (2021). The assessment of big data adoption readiness with a technology–organization–environment framework: a perspective towards healthcare employees. Sustainability.

[59] Maroufkhani P. (2020). Big data analytics adoption: determinants and performances among small to medium-sized enterprises. Int. J. Inf. Manag..

[60] Pan Y. (2022). The adoption of artificial intelligence in employee recruitment: the influence of contextual factors. Int. J. Hum. Resour. Manag..

[61] Maroufkhani P., Wan Ismail W.K., Ghobakhloo M. (2020). Big data analytics adoption model for small and medium enterprises. Journal of Science and Technology Policy Management.

[62] Rogers E.M., Singhal A., Quinlan M.M. (2014). An Integrated Approach to Communication Theory and Research.

[63] Gangwar H. (2018). Understanding the determinants of big data adoption in India: an analysis of the manufacturing and services sectors. Inf. Resour. Manag. J..

[64] Jarrar Y., Awobamise A., Sellos P. (2020). Technological Readiness Index (TRI) and the intention to use smartphone apps for tourism: a focus on inDubai mobile tourism app. International Journal of Data and Network Science.

[65] Anjum N., Islam M.A. (2020). Employees' behavioral intention to adopt e-HRM system-an approach to extend technology acceptance model. Int. J. Acad. Res. Account. Finance. Manag. Sci..

[66] Chen S.-C. (2021). Assessing determinants of continuance intention towards personal cloud services: Extending utaut2 with technology readiness. Symmetry.

[67] Awa H.O., Ukoha O., Igwe S.R. (2017). Revisiting technology-organization-environment (TOE) theory for enriched applicability. Bottom Line.

[68] Jahanshahi A., Brem A. (2017). Sustainability in SMEs: Top management teams behavioral integration as source of innovativeness. Sustainability.

[69] Sanders N.R. (2008). Pattern of information technology use: the impact on buyer–suppler coordination and performance. J. Oper. Manag..

[70] Walker J.H., Saffu K., Mazurek M. (2016). An empirical study of factors influencing e-commerce adoption/non-adoption in Slovakian SMEs. J. Internet Commer..

[71] Vaishnavi V., Suresh M., Dutta P. (2019). Modelling the readiness factors for agility in healthcare organization: a TISM approach. Benchmark Int. J..

[72] Saeed I., Juell-Skielse G., Uppström E. (2012). Cloud enterprise resource planning adoption: Motives & barriers. Advances in Enterprise Information Systems II.

[73] Amini M. (2014). Development of an instrument for assessing the impact of environmental context on adoption of cloud computing for small and medium enterprises. Australian Journal of Basic and Applied Sciences (AJBAS).

[74] Baker J.J.I.s.t. (2012).

[75] Chen H., Chiang R.H., Storey V.C.J.M.q. (2012).

[76] Chen Y., Alspaugh S., Katz R.J.a.p.a. (2012).

[77] Dash S. (2019). Big data in healthcare: management, analysis and future prospects.

[78] Kalema B.M., Mokgadi M.J.P. (2017).

[79] Kalema B.M., Motsi L., Motjolopane I.M. (2016). Utilizing IT to enhance knowledge sharing for school educators in developing countries.

[80] Kruse C.S. (2016). Challenges and opportunities of big data in health care: a systematic review.

[81] Lemekwane M. (2016).

[82] Ghaleb E.A. (2023). Assessing the big data adoption readiness role in healthcare between technology impact factors and intention to adopt big data.

[83] Haddad A. (2018). The impact of intention of use on the success of big data adoption via organization readiness factor.

[84] Mukherjee S., Chittipaka V., Mohan Baral M. (2022). Pattern Recognition and Data Analysis with Applications.

[85] Ibeneme S. (2021). BMC Proceedings.

[86] Muhammad N.K., Management K. (2022). A conceptual framework for big data analytics adoption towards organization performance in Malaysia.

[87] Alharbi F. (2016).

[88] Hashim H. (2021). Conceptualizing the relationship between Big Data Adoption (BDA) factors and Organizational Impact (IO).

[89] Hou J. (2023).

[90] Wang Y. (2018).

[91] Frimpong E. (2023).

[92] Egwuonwu A. (2023). Drivers of big data analytics' adoption and Implications of management decision-making on big data adoption and firms' financial and nonfinancial performance. Evidence From Nigeria's Manufacturing and Service Industries.

[93] Salman M.S. (2022). Assessing the big data analytics readiness based on technology-organization-environment (TOE) framework of Malaysian libraries. Descriptive Analysis.

[94] Nguyen G.T. (2022). Readiness of SMEs for adopt big data: an empirical study in vietnam.

[95] Ngusie H.S. (2022). Healthcare providers' readiness for electronic health record adoption: a cross-sectional study during pre-implementation phase.

[96] Alzghaibi H. (2023). Assessing primary health care readiness for large-scale electronic health record system implementation: Project team perspective.

[97] Stenberg L., Nilsson S. (2020).

[98] Habimana Y. (2020). An Adoption model for a big data analytics system for improving healthcare services in Burundi's public hospitals.

[99] Sun S. (2018). Understanding the factors affecting the organizational adoption of big data.

[100] Manohar P. (2020).

[101] Akal T.D. (2019). ICT Unbounded, Social Impact of Bright ICT Adoption: IFIP WG 8.6 International Conference on Transfer and Diffusion of IT, TDIT 2019, Accra, Ghana, June 21–22, 2019, Proceedings.

