# Supplementary material for: Readiness of big health data analytics by technology-organization-environment (TOE) framework in Ethiopian health sectors
Source: Heliyon. 2024 Sep 27;10(19):e38570. doi: 10.1016/j.heliyon.2024.e38570 (PMC11470786; doi:10.1016/j.heliyon.2024.e38570)
Supplement: Multimedia component 1 [file mmc1.docx]

## **Informed consent statement**

To assess **Readiness of Big Health Data Analytics by Using the Technology-Organization-Environment (TOE) Framework in Ethiopian Health Sectors: A Structural Equation Modeling Approach.**

Hello. My name is _____________________I am here on behalf of Bayou Tilahun (MPH) has ethical clearance from Debre Markos University to conduct the research on the above topic. Your participation in the study is voluntary to participate in this study and your anonymous answers will be used only for research purposes. To effectively attain the purpose of the research, I request you to give a genuine response to each question. There are questions for you to complete and there is no need to put your name on the questionnaire; no individual responses will be reported. Your answers are completely confidential. It is your full right to refuse, to answer any or all of the questions. If you don't want to participate you can tick “No” leave the questionnaire. Study questionnaires will take a maximum of 25 minutes.

I understand that the findings of this research will be disseminated to Hospital management and decision-makers that will be useful as input for intervention design.

Agreed to participate in the study: Yes No

**For any further questions, contact the investigator**

Name: -Bayou Tilahun

Email:- ([bayutilahun5@gmail.com](mailto:bayutilahun5@gmail.com) )

Phone no: - 0936210997

**Thank you**

| **Socio-demographic related questions (encircle your choice)** | | | |  |
| --- | --- | --- | --- | --- |
| **SN** | **QUESTION** | **Response option** | **Code** | **Skip** |
| 1 | Gender | 1. Male 2. Female |  |  |
| 2 | Age | _______year(s) |  |  |
| 3 | Educational status | 1. Medical doctors 2. Master’s degree 3. Bachelor degree 4. Diploma 5. Others …………(specify) |  |  |
| 4 | Work experience(years) | …………(years) |  |  |

**By using the rating scale from 1-5 where; 1 = strongly disagree, 2 =Disagree, 3 = Neutral, 4= Agree, 5 = strongly agree. Indicate your level of agreement or disagreement on the following constructs to measure big health data readiness**

| **To Assess** **Readiness of Big Health Data Analytics by Using the Technology-Organization-Environment (TOE) Framework in Ethiopian Health Sectors: A Structural Equation Modeling Approach.**  **By using the rating scale from 1-5 where; 1 = strongly disagree, 2 =Disagree, 3 = Neutral, 4= Agree, 5 = strongly agree. Indicate your level of agreement or disagreement on the following constructs to measure big health data readiness** | | | | | | | | | | | | | |
| --- | --- | --- | --- | --- | --- | --- | --- | --- | --- | --- | --- | --- | --- |
|  | **Constructs** | | Items | | Strongly disagree(1) | | Disagree(2) | | Neutral(3) | | Agree(4) | | Strongly agree(5) |
| **Technological context** | **Complexity (CX)** | |  | | 1 | | 2 | | 3 | | 4 | | 5 |
|  | BD allows me to manage business operations in an efficient way. | | CX1 | | 1 | | 2 | | 3 | | 4 | | 5 |
|  | The use of BD is frustrating. | | CX2 | | 1 | | 2 | | 3 | | 4 | | 5 |
|  | The skills needed to improve and use the new technologies are easy for me. | | CX3 | | 1 | | 2 | | 3 | | 4 | | 5 |
|  | The use of BD requires a lot of mental effort. | | CX4 | | 1 | | 2 | | 3 | | 4 | | 5 |
|  | **Compatibility (CT)** | | | | | | | | | | | | |
|  | The use of BD is compatible with my healthcare corporate culture and value system. | CT1 | | 1 | | 2 | | 3 | | 4 | | 5 | |
|  | The use of BD will be compatible with existing hardware and software. | CT2 | | 1 | | 2 | | 3 | | 4 | | 5 | |
|  | BD is easy to use and manage | CT3 | | 1 | | 2 | | 3 | | 4 | | 5 | |
|  | BD is compatible with existing emerging technologies | CT4 | | 1 | | 2 | | 3 | | 4 | | 5 | |
|  | **Optimism (OP)** | | | | | | | | | | | | |
|  | New technologies contribute to a better quality of life. | OP1 | | 1 | | 2 | | 3 | | 4 | | 5 | |
|  | Technology gives me more freedom of mobility | OP2 | | 1 | | 2 | | 3 | | 4 | | 5 | |
|  | Technology gives people more control over their daily lives | OP3 | | 1 | | 2 | | 3 | | 4 | | 5 | |
|  | Technology makes me more productive in my personal life. | OP4 | | 1 | | 2 | | 3 | | 4 | | 5 | |
|  | Technology makes me more efficient in my occupation. | OP5 | | 1 | | 2 | | 3 | | 4 | | 5 | |
| Organizational context | **Top Management support (TMS)** | | | | | | | | | | | | |
|  | Top management supports plans to adopt the big data. | **TMS1** | | 1 | | 2 | | 3 | | 4 | | 5 | |
|  | Top management will support the implementation of  BD adoption. | **TMS2** | | 1 | | 2 | | 3 | | 4 | | 5 | |
|  | Top management support is important to provide the resources for the company to adopt big data. | **TMS3** | | 1 | | 2 | | 3 | | 4 | | 5 | |
|  | The healthcare management is willing to take risks (financial and organizational) involved in the adoption of big data. | **TMS4** | | 1 | | 2 | | 3 | | 4 | | 5 | |
|  | The firm size compatible with the adoption of big data. | **TMS5** | | 1 | | 2 | | 3 | | 4 | | 5 | |
|  | **Financial support (FS)** | | | | | | | | | | | | |
|  | Financial support is important for purchasing new technology equipment. | **FS1** | | 1 | | 2 | | 3 | | 4 | | 5 | |
|  | Financial support for the BD technology will strengthen the current system infrastructure in healthcare | **FS2** | | 1 | | 2 | | 3 | | 4 | | 5 | |
|  | Financial support will help to better secure the patient’s data. | **FS3** | | 1 | | 2 | | 3 | | 4 | | 5 | |
|  | My company has the financial resources to purchase the hardware and software required for technologies. | **FS4** | | 1 | | 2 | | 3 | | 4 | | 5 | |
|  | **Training (TR)** | | | | | | | | | | | | |
|  | Training on the BD usage is meeting my requirements | **TR1** | | 1 | | 2 | | 3 | | 4 | | 5 | |
|  | Training on BD usage ensures that employees have received the appropriate training. | **TR2** | | 1 | | 2 | | 3 | | 4 | | 5 | |
|  | Training on BD usage is adequate for all involved staff. | **TR3** | | 1 | | 2 | | 3 | | 4 | | 5 | |
|  | All users have been trained in basic technology skills in the healthcare system. | **TR4** | | 1 | | 2 | | 3 | | 4 | | 5 | |
| Environmental context | **Government IT policies (GITP)** | | | | | | | | | | | | |
|  | Government IT policy can attract more foreign investors to invest in sustainable businesses. | **GITP1** | | 1 | | 2 | | 3 | | 4 | | 5 | |
|  | Government IT policy can encourage sustainable technology usage | **GITP2** | | 1 | | 2 | | 3 | | 4 | | 5 | |
|  | Government IT policy can improve sustainable technology efficiency. | **GITP3** | | 1 | | 2 | | 3 | | 4 | | 5 | |
|  | Government IT policy can educate sustainable technology in Ethiopia on the benefits of sustainable technology | **GITP4** | | 1 | | 2 | | 3 | | 4 | | 5 | |
|  | There is a lack of security rules, IT policies, and privacy laws | **GITP5** | | 1 | | 2 | | 3 | | 4 | | 5 | |
|  | **Government lows and legislations (GLAL)** | | | | | | | | | | | | |
|  | The laws and regulation that exist nowadays are sufficient to protect the use of big data. | **GLAL1** | | 1 | | 2 | | 3 | | 4 | | 5 | |
|  | The government drives the use of the BD through incentive programs | **GLAL2** | | 1 | | 2 | | 3 | | 4 | | 5 | |
|  | The company requires maintaining the regulatory environment in the use of big data | **GLAL3** | | 1 | | 2 | | 3 | | 4 | | 5 | |
|  | The laws and regulations of the government support BD initiatives and implementation | **GLAL4** | | 1 | | 2 | | 3 | | 4 | | 5 | |
|  | Government laws and regulations can provide a better process for adopting technologies | **GLAL5** | | 1 | | 2 | | 3 | | 4 | | 5 | |
| **BHD Readiness** | **BD Readiness (BDR) in Healthcare Sector** | | | | | | | | | | | | |
|  | The healthcare management understands how they can be used in the healthcare sector | **BDR1** | | 1 | | 2 | | 3 | | 4 | | 5 | |
|  | The healthcare IT infrastructure is good (internet service/devices) and can be used for big data | **BDR2** | | 1 | | 2 | | 3 | | 4 | | 5 | |
|  | The healthcare management already promoted the usage of the BD to the staff very well | **BDR2** | | 1 | | 2 | | 3 | | 4 | | 5 | |
|  | The healthcare staffs have the right skills to work with big data. | **BDR3** | | 1 | | 2 | | 3 | | 4 | | 5 | |
|  | The healthcare IT department and the healthcare management have the right skills to lead the healthcare transformation, and they give very good support to help the staff. | **BDR4** | | 1 | | 2 | | 3 | | 4 | | 5 | |
